# The physiological and physical benefits of two types of concurrent training: a randomized controlled trial

**DOI:** 10.1186/s13102-023-00798-x

**Published:** 2024-01-02

**Authors:** Umut Canli, Monira I. Aldhahi

**Affiliations:** 1 Sports Science Faculty, Tekirdag Namik Kemal University, Tekirdag, Turkey; 2 Department of Rehabilitation Sciences, College of Health and Rehabilitation Sciences, Princess Nourah bint Abdulrahman University (PNU), P.O. Box 84428, Riyadh, 11671 Saudi Arabia

**Keywords:** Aerobic training, Endurance, Anaerobic work, Strength, Training protocols

## Abstract

**Background:**

It is widely acknowledged that aerobic exercise and strength training are crucial components of most workout programs. However, there is no consensus as to whether the effectiveness of exercises is affected by the sequence in which they are performed. Therefore, the overarching aim of the study was to understand the optimal order of two types of concurrent training program for 13 weeks by comparing the effectiveness of the training on body composition, predicated maximal oxygen uptake (VO_2max_), dynamic respiratory parameters and muscle strength in healthy middle-aged people.

**Methods:**

Thirty-three middle-aged individuals, who were categorized as moderately active based on their responses to International Physical Activity Questionnaires, underwent random allocation. The participants were randomly assigned into two groups: the Strength Training followed by Aerobic Training group (SAG, n = 16) and the Aerobic Training followed by Strength Training group (ASG, n = 17). Body composition, aerobic endurance, respiratory parameters, and upper and lower strength were assessed at baseline and after (post-test) a 13-week intervention. The chi-square test and the independent t-test were used to compare sociodemographic variables between the groups. A 2 × 2 analysis of variance (ANOVA) with repeated measures (group x measurement) was conducted. The study was retrospectively registered on clinicaltrials.gov in May of 2023 (clinicaltials.gov identifier: NCT05862415; in 04/25/2023).

**Results:**

Findings showed no significant differences between the group in the VO_2max_, FVC or FEV1 (F = 1.122, 0.028, 0.06, 2.483; *p* > 0.05, respectively). Intragroup analysis revealed changes in PEF compared to baseline in the ASG (F = 5.895; *p* < 0.05). Increases were observed in all strength parameters for both training programs.

**Conclusions:**

The concurrent training effect on muscle composition, oxygen consumption and muscle strength specifically 1RM, in middle-aged individuals are equivocal, regardless of the exercise order. The results indicate that both exercise sequences can elicit similar benefits in terms of cardiovascular fitness, muscular strength, and endurance. This lack of difference suggests that the order of exercise does not play a significant role in determining the effectiveness of the workout or the subsequent physiological adaptations.

**Clinicaltials.gov identifier:**

NCT05862415. Date of registration: 04/25/2023

## Background

Globally, the total population of middle-aged adults is rapidly increasing towards older adults. [WHO, 2015]. The expansion of aging research is evident, particularly in western nations, due to the increase in the number of older adults [1]. As a consequence, the aging process has emerged as a noteworthy interest for both national and international health institutions [2]. Aging causes a progressive decline in aerobic fitness, strength, and muscle mass [3]. Declines in aerobic fitness, strength, and muscle mass are linked to a higher incidence of type 2 diabetes [4], cardiovascular disease [5], and risk of falls [6]. According to studies by Snowden et al. [7] and Sui et al. [8] indicate that decreased cardiorespiratory fitness due to aging is the primary factor linked to hospitalization, morbidity, and mortality. In the pathophysiology of functional impairment and frailty, sarcopenia and dynapenia are crucial factors as age increases [9, 10].

Previous research has shown that the level of physical fitness in middle age can predict physical performance in later life, implying that earlier training interventions at 50 years can result in positive long-term effects [11]. Maintaining a high level of physical fitness is critical in both middle-aged and older adults. The mechanisms underlying age-related changes in physical fitness and motor control are multifactorial but not fully understood [12] Exercise training is widely recommended as a safe and effective way to prevent the accelerated decline in cardiorespiratory fitness and muscular strength that occurs with age. This can help maintain physical health and function, as well as slow the progress of chronic diseases. Moreover, the type of exercise performed influences the adaptations induced by exercise training [13]. Concurrent training (CT) methods, in particular, have become popular in recent years as an exercise strategy. In older populations, a combination of strength and aerobic training (i.e., concurrent training) appears to be the most effective strategy for improving both neuromuscular and cardiorespiratory functions [14–17].

A meta-analysis recently conducted on healthy adults demonstrated no significant difference in aerobic capacity between the concurrent training groups, while also offering comparable efficacy to resistance training in enhancing muscular strength [18]. Additionally, findings from a separate meta-analysis suggest that CT has no effect on maximal strength or hypertrophy [19]. Nevertheless, Jones and colleagues have observed that performing endurance exercises prior to strength exercises can reduce resistance training performance [20]. The influence of concurrent strength and endurance training can be affected by various factors such as exercise method, sequence, duration, frequency, intensity, and subject characteristics, which has been shown through research in this field [21, 22]. The order of strength and endurance training in a concurrent training session and its impact on pulmonary profile, cardiopulmonary fitness, and strength, particularly among healthy middle-aged adults, remains unclear. The existing literature consists of studies with heterogeneous samples, encompassing a wide range of ages and levels of physical activity, which accounts for discrepancy in the findings. Therefore, the purpose of this research study is to compare the effect of two training protocols on healthy, active middle-aged adults over the course of 13 weeks. The two protocols differed in their order of concurrent training, which combines aerobic and resistance exercises. The study aims to understand the impact of these protocols on body composition, upper and lower body strength, cardiovascular fitness (VO_2max_), and dynamic respiratory parameters. We hypothesized that regardless of the order in which the concurrent training protocols were implemented, there would be no significant difference in the outcomes between the two protocols.

## Methods

### Study design and participants

A randomized controlled trial was conducted to investigate the effect of two different concurrent training protocols on physiological parameters and physical performance among healthy middle-aged people over a 13-week period. A total of 33 healthy middle-aged (age = 42.93 ± 8.86 years), were randomly assigned (1:1), using a randomized block design to concurrent training groups: (1) a group that received concurrent training of strength followed by aerobic [SAG, n = 16]; (2) a group that received aerobic followed by Strength training [ASG, n = 17].

**Fig. 1:**
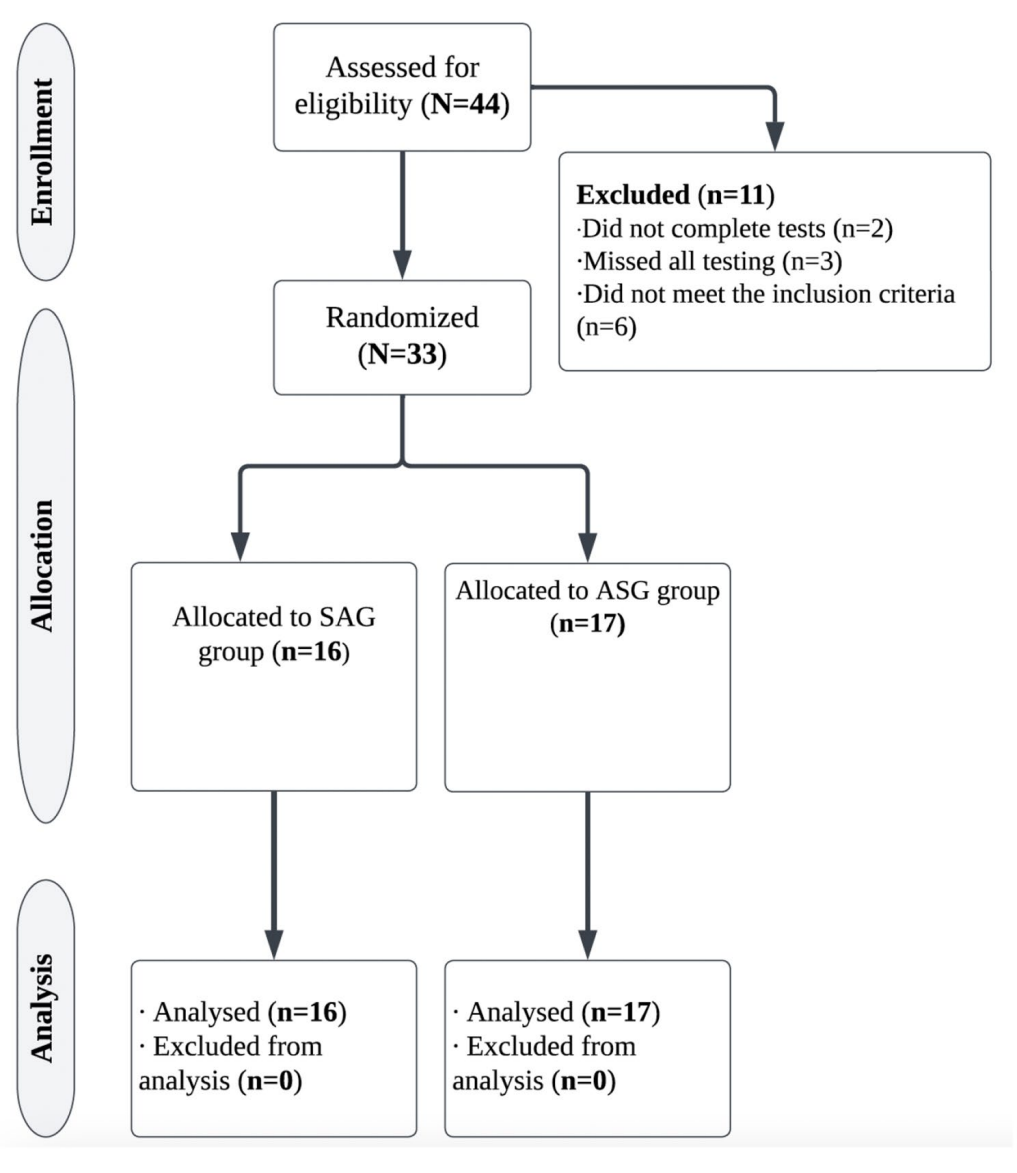
Study flow diagram

Figure 1 displays the study flow diagram. The study inclusion criteria were: (a) being over the age of 40; (b) not having cardiovascular or neuromuscular disorders; (c) not having orthopedic disorders; and (d) not having neurologic disorders. The exclusion criteria consisted of the following: (a) patients with artificial prostheses; (b) individuals who had participated in a structured training program; (c) individuals with symptoms deemed by a medical examiner to be sufficient for exclusion; (d) individuals with diseases that make exercise impossible or require special care (e.g. coronary artery disease, thrombosis, moderate or severe bone disease, and lung or renal disease); and (e) individuals with diseases that require daily use of drugs affecting athletic performance to avoid any influence on fitness measures. After fulfilling the inclusion criteria, participant consent forms were obtained prior to study entry, in accordance with the Helsinki Declaration and subsequent amendments [23]. In addition, all methods and procedures have been approved by Tekirdag Namik Kemal University’s Scientific Research and Publication Ethics Committee granted ethical approval (Protocol No: 2021.275.11.19) and the study was retrospectively registered on clinicaltrials.gov in May of 2023 (clinicaltials.gov identifier: NCT05862415).

### Study procedures

This is a parallel group randomized controlled trial study. All measurements were taken over the course of two sessions. During the initial session, the demographics of the subjects were recorded, including age, gender, medical history (chronic health status number), smoking habits, and other characteristics. The seven-day physical activity recall questionnaire (7 Day IPAQ) was utilized to estimate the subjects’ physical activity level. Individuals were categorized as having a moderate level of physical activity if they met any of the following criteria: Engaged in 3 or more days of vigorous-intensity activity and/or walking for at least 30 min per day; or Engaged in 5 or more days of moderate-intensity activity and/or walking for at least 30 min per day; or engaged in 5 or more days of any combination of walking, moderate-intensity, or vigorous-intensity activities, achieving a minimum total physical activity of at least 600 MET-minutes per week. Before starting with the training protocols, all anthropometric measurements were taken, and physical tests were conducted. The testing sessions were conducted with a 72-hour interval between the start of the program and baseline assessment. In the initial visit, participants filled out a questionnaire where they provided demographic details and completed the 7-Day IPAQ short form. After this, a series of evaluations were conducted, which included measurements of body anthropometric measures and grip strength tests, followed by the 1RM strength assessments. In the second visit, participants underwent spirometric tests and a 1-mile run-walk test. All tests were conducted at the same time of day, between 17:30 and 19:30, to control for the impact of circadian rhythms. The following testing procedures were implemented:

#### Body composition assessment

Height (m) was measured using a Mesilife 13,539 brand portable stadiometer with accuracy of 0.1 cm. A stabile eight-polar tactile electrode bioelectrical impedance analyzer (Tarti Fast, Japan) was used to measure body mass (kg), fat percentage, and skeletal muscle mass (kg). This bioelectrical impedance analyzer’s validity has previously been reported [24]. Body mass index (BMI) (kg/m^2^) = was calculated by dividing body mass (kg) by body height squared (m^2^).

#### Physical activity level

The short form of International Physical Activity Questionnaire (IPAQ) was used to assess the physical activity status [25]. The questionnaire inquired about the duration and frequency individuals allocate to vigorous-intensity exercise, moderate exercise, walking, and sitting during the day. The metabolic equivalents (i.e., METs/week) related to vigorous PA, moderate-intensity PA, and walking, and total time of PA performed were calculated using guidelines for data analysis [26].

#### Predicated maximal oxygen uptake (VO_2max_)

1-mile walk-run performance test was used. The aim of this test is to cover a distance of one mile in the shortest possible time. Participants can alternate between walking and running as desired; however, they should be encouraged to cover the distance as quickly as feasible. The time elapsed to cover a mile distance, measured in minutes and seconds. During the testing and recovery, the heart rate of the participants was monitored with a heart rate sensor (Polar Verity Sense; Kempele, Finland). Polar Verity Sense can be connected to a sports watch or app via Bluetooth®, ANT+, and internal memory to instantly track your training or view the data after training. As a result, the participant’s time for completing the 1-mile test was tracked using a smart phone GPS system. The validity of the device has been reported previously [27]. The participants VO_2max_ values were calculated using the formulas below:

Male (VO_2max_) = 108.844 - 0.1636W - 1.438T - 0.1928 H

Female (VO_2max_) = 100.5 - 0.1636W - 1.438T - 0.1928 H

W = Weight in kg, T = Time for the one-mile run and H = Heart rate at the end of the run [28].

#### Dynamic respiratory parameters

All spirometry measurements were taken in accordance American Thoracic Society (ATS) and European Respiratory Society (ERS) [29]. We used a spirometer device (Minispir®, PC-based Spirometer with Oximetry option, Italy) to take measurements from the participants. Participants were instructed to hold and sealed the lip around the mouthpiece then breathe normally twice and then exhale the maximally filled lungs with a maximal breath through the mouthpiece orifice. Each test was repeated twice, and the best results were recorded for each participant. We measured the Forced Vital Capacity (FVC), Forced Expiratory Volume in One Second (FEV1), and Peak Expiratory Flow in this procedure (PEF).

#### Grip strength

A handgrip dynamometer was used to measure the strength of the hand and forearm muscles (in kilograms) (Tanita Handgrip Meter, RM40, East Malaysia). The test was carried out with the elbow fully extended and the forearm in a neutral position. At 1-minute intervals, three measurements were taken on the subjects’ dominant side. The participants were instructed to told the handgrip of the device tightly for 3 s [30]. For statistical analysis, the highest scores from three measurements were taken.

#### 1-repetition maximum strength test

The 1 repetition maximum (1RM) test, an indirect and simple test, can be effectively and safely administered. It is feasible to calculate the 1RM value using the calculations based on the maximum repetition limit between 2 and 20 for any weight. In order to determine the 1RM values of the study participants, indirect 1RM bench press, leg press, long pulley, leg extension, and overhead press were used. 1RM tests were performed using a weight machine (Technogym Selection 900). The formula for calculating 1RM indirectly is given below [31].

1RM= (Lifted Weight) / [1.0278 ? (Repetition*0.0278)]

#### Training program

**Fig. 2:**
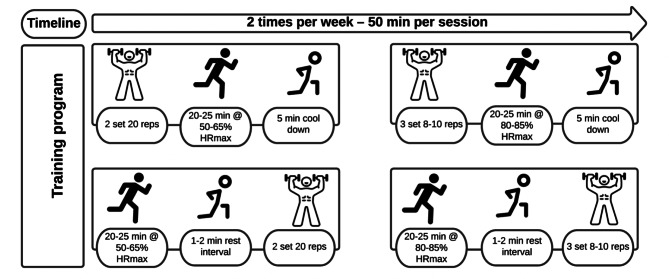
An illustrative scheme of training program across groups

Training sessions were conducted twice a week for 13 weeks, either indoors or outdoors at an athletic field pavilion. Each session started with a 5- to 7-minute warm-up, consisting of low-intensity walking and running, and dynamic mobility exercises. Active recovery of 4- to 5-minute was based on stretching and relaxation exercises. Sessions lasted approximately 50 min (warm-up and cold down included). A detailed description of the 13-week training program is reported in Fig. 2.

All training sessions were supervised by 8 experienced personal trainers. Paired groups were formed according to the similarity between the strength and aerobic fitness levels of the participants. The circular training method was used in the resistance training phase. During the aerobic training phase, a series of movements from moderate intensity to vigorous intensity was performed. The step loading periodization principle was taken into account in both resistance training and aerobic exercises.

The RT lasted approximately 20–25 min per session and included two sets of 8–20 repetitions, with a rest interval of 1–2 min [32]. The assessing of the Repetition Maximum (RM) involves opting for a range of RM targets. Subsequently, participants execute each exercise with varying levels of resistance until they achieve the predetermined RM goal. The resistance training program was performed using the circuit training method in which two or three sets were performed. Intensity was measured with the Borg CR-10 scale [33], initially starting at 2 points and progressively increasing to five points weekly as described in Table 1. The RT exercises included: squat, barbell bent-over row, overhead press, plank, lateral pull down, triceps push down, barbell curl, leg extension, leg curl, lunge, barbell bench press, crunches etc. Especially in the first 4th weeks of the training protocol, it was preferred to use hydraulic and roller system fitness machines in resistance exercises. After the 5th week, free weights such as dumbbells and barbells were started to be used as the exercise loads progressed. All the training sessions were supervised by researcher who conducted the exercise sessions and assured the safety and the correct execution of the movement.

**Table 1:** Periodization of strength training

Weeks	Intensity (Borg a Category-Ratio [CR-10])	Repetitions	Average Intensity (Weight lift)
1–2	2	15–20	10–25
3–4	3	15	26–40
5–7	4	12–15	41–55
8–10	5	10–12	56–75
11–13	6	8–10	76–95
11–13	6	8–10	

Aerobic exercise training was designed to include different aerobic training protocols. Intensity was established between 50 and 65% and 80–85% of HRmax for both groups (SAG and ASG). The HRmax was estimated using Tanaka et al. [34] equation (i.e. (208 – age) ∗ 0.7). The heart rate variables of the participants during aerobic exercise were determined using the heart rate sensor. In line with the step loading periodization principle; an aerobic training program was designed from moderate to vigorous intensity, with HRmax varying between 50 and 85% from the 1st week to the 13th week of the participants. The aerobic training program lasted approximately 20–25 min. Aerobic training includes walking and running periods and was carried out on a 400 m outdoor track. In periodization, especially 1st-4th. walking, jogging and running-based exercises were preferred between weeks. In the following weeks, in addition to these exercises, more intense exercises were added to the training program (rope jumping, jumping jack, burpee, vertical jumping, broad jumping, sliding, footwork etc.). The training strategy was the same in both groups.

In SAG, the order of exercises began with strength training followed by aerobic training, whereas in ASG, aerobic training was conducted before engaging in strength training. Figure 2 highlights the discrepancy in the exercise order between the two groups.

### Statistical analyses

The data were analysed with the statistical program SPSS v.18.0 for Windows (SPSS Inc., Chicago, USA), and the significance level was set at *p* < 0.05. Before conducting any analysis, all data were checked for normality using the Shapiro-Wilk test and homogeneity with the Levene’s test, respectively. Descriptive statistics are presented as mean and standard deviation (SD). The chi-square test and the independent t-test were used to compare sociodemographic variables between the groups. A repeated measures 2 × 2 analysis of variance (ANOVA) (group x measurement) was conducted. Before performing ANOVA, the necessary conditions for analysis were checked. Box’s Test of Equality of Covariance Matrices was used to evaluate whether there were any significant differences in the covariances between the measurement groups for pairwise combinations of the groups. In addition, the magnitudes of the differences between values were interpreted using the partial eta squared effect size. The partial eta squared coefficient (η_p_^2^) was presented as it indicates how much of the variance of the dependent variable is explained by the independent variable [35].

## Results

At baseline, there were no significant differences between-group (*p* > 0.05) in anthropometric characteristics, sex distribution, physical activity level, or sitting time (Table 2). The results of the two-way ANOVA showed that there were no statistically significant differences between the two groups with regards to body composition parameter (body mass, BMI, fat mass, muscle mass) as part of the group-time interaction analysis (F) = 0.214; 0.278; 1.146; 0.379; *p* > 0.05), which can be seen in Table 3.

**Table 2:** Characteristic of participants

Variable	SAG (*n* = 16)	ASG (*n* = 17)	*p-value*
	**Mean ± SD**	**Mean ± SD**	
Age (y)	44.61 ± 6.22	41.35 ± 10.73	0.29
Height (cm)	162.97 ± 6.82	161.71 ± 9.02	0.65
Body mass (kg)	70.70 ± 14.85	70.90 ± 15.16	0.97
Body mass index (kg.m^− 2^)	26.65 ± 5.52	27.07 ± 5.17	0.82
Sex, * *n* (%)			
Male	5 (31.2)	4 (23.5)	0.61
Female	11 (68.8)	13 (76.5)	
PA (MET-min/week)	740.18 ± 758.64	639.70 ± 654.32	0.68
Sitting time (hours)	7.81 ± 3.97	9.00 ± 3.92	0.39

^*Chi−square test; PAL = physical activity; SD = standard deviation^

**Table 3:** Body composition parameters before (pre-test) and after (post-test) a 13-week training

Variables	Groups	Pre-test	Post-test	F	*p-value* (group x time)	*η*_*p*_^*2*^
		mean ± SD	mean ± SD			
Body mass (kg)	SAG	70.70 ± 14.85	69.08 ± 14.20	0.214	0.64	-
	ASG	70.90 ± 15.16	69.59 ± 14.15			
BMI (kg.m^− 2^)	SAG	26.65 ± 5.52	26.06 ± 5.25	0.278	0.60	-
	ASG	27.07 ± 5.17	26.58 ± 4.90			
Fat mass (%)	SAG	29.60 ± 8.21	27.35 ± 8.20	1.146	0.29	-
	ASG	32.71 ± 6.79	31.00 ± 6.59			
Muscle mass (kg)	SAG	46.32 ± 8.19	47.02 ± 7.53	0.379	0.54	-
	ASG	44.99 ± 9.44	45.44 ± 9.19			

^SAG = strength+aerobic group; ASG = aerobic+strength group; BMI = body mass index; SD = standard deviation; ηp2 = partial eta squared^

It was observed that there were significant increases in the values of peak expiratory flow (PEF) in the ASG (F = 5.895; η_p_^2^_=_ 0.160; *p* < 0.05; η_p_^2^_=_ 0.160), leading to the conclusion that ASG has a significant effect on increasing the PEF value (Table 4). However, participating in either the SAG or ASG groups had no significant influence on VO_2Max_, FVC or FEV1 (post-test–pre-posttest difference, F = 1.122, 0.028, 0.06, 2.483; *p* > 0.05, respectively).

**Table 4:** VO_2max_ and dynamic respiratory parameters before and after a 13-week training

Variables	Groups	Pre-test	Post-test	F	*p* (group x time)	*η*_*p*_^*2*^
		mean ± SD	mean ± SD			
VO_2max_ (ml/kg/min)	SAG	44.21 ± 5.84	43.89 ± 5.02	1.122	0.29	-
	ASG	42.43 ± 4.43	40.67 ± 5.61			
FVC (L)	SAG	4.14 ± 0.85	4.01 ± 0.73	0.028	0.86	-
	ASG	4.02 ± 1.56	3.84 ± 1.34			
FEV_1_ (L/s)	SAG	3.02 ± 0.70	2.72 ± 0.74	2.483	0.12	
	ASG	2.60 ± 1.09	2.68 ± 0.94			
PEF (L/s)	SAG	5.90 ± 1.92	5.05 ± 1.94	5.895	0.02	0.160
	ASG	4.78 ± 2.25	5.15 ± 2.09			

^*p* < 0.05*; SAG = strength+aerobic group; ASG = aerobic+strength group; FVC = forced vital capacity; FEV1 = forced expiratory volume; PEF = peak expiratory flow; SD = standard deviation; ηp2 = partial eta squared^

When the pre-test and post-test scores in both SAG and ASG were examined, increases were observed in all strength parameters (Table 5). The results of the 2 × 2 ANOVA test indicated that the increases did not result in any significant difference between the SAG and ASG groups in terms of the participants’ strength values (F = 0.135; 0.783; 0.814; 0.284; 0.280; 0.580; *p* > 0.05, Respectively).

**Table 5:** Strength parameters before and after a 13-week training

Variables	Groups	Pre-test	Post-test	F	*p-value* (group x time)	*η*_*p*_^*2*^
		mean ± SD	mean ± SD			
Hand-grip strength (kg)	SAG	36.73 ± 6.84	36.76 ± 5.94	2.362	0.13	-
	ASG	32.35 ± 7.15	33.68 ± 9.15			
1RM bench press (kg)	SAG	31.40 ± 13.28	40.28 ± 16.45	0.077	0.78	-
	ASG	29.27 ± 14.47	38.98 ± 16.23			
1RM leg press (kg)	SAG	90.77 ± 62.03	141.90 ± 63.81	0.056	0.81	-
	ASG	89.53 ± 46.60	145.89 ± 79.34			
1RM long pulley (kg)	SAG	33.88 ± 11.61	41.03 ± 10.94	1.190	0.28	-
	ASG	31.18 ± 10.66	34.74 ± 11.85			
1RM leg extension (kg)	SAG	42.65 ± 13.93	56.93 ± 22.98	1.651	0.28	-
	ASG	44.52 ± 19.51	53.57 ± 20.28			
1RM overhead press (kg)	SAG	24.93 ± 10.23	32.92 ± 11.03	0.313	0.580	-
	ASG	21.53 ± 9.42	27.88 ± 12.96			

^SAG = strength+aerobic group; ASG = aerobic+strength group; 1RM = one−repetition maximum; SD = standard deviation; ηp2 = partial eta squared^

## Discussion

This study aimed to examine the impact of two different sequence of concurrent training programs on body composition, muscle strength, fitness and Pulmonary functioning profile. The results indicate that the concurrent training irrespective of the exercise order yielded comparable improvements in all body composition parameters. In the SAG, body mass decreased by 2.30%, BMI by 2.22%, and fat mass by 7.60%, while muscle mass increased by 1.51%. In the ASG, body mass decreased by 1.85%, BMI by 1.82%, fat mass by 5.23%, while muscle mass increased by 1%. These finding aligned with study by Salamat et al. [36] in young men which revealed a decrease in body fat percentage for both concurrent training groups after 8 weeks. However, the findings were not statistically significant. On the other hand, the results of a previous study on the effects of moderate frequency two types of concurrent training protocols did not observe significant changes in body fat percentage, total fat mass, or abdominal fat mass in the two groups at either week 12 or week 24 in aged 30 healthy men [19]. However, both concurrent types’ s changes in body composition were so similar [37]. It has been determined that there are positive developments in body composition in different types of concurrent training methods performed to different populations and healthy or unhealthy individuals. In addition, it was determined that the order of resistance or aerobic training was not important.

The current study presented that both concurrent training protocols lead to similar improvements in VO_2max_ for healthy middle-aged people. The lack of significant changes or differences in VO_2max_ (maximal oxygen uptake) between the two groups could be attributed to some potential factors. In concurrent training, the order in which aerobic and strength training are performed can influence their respective adaptations. Some studies suggest that performing aerobic training before strength training may interfere with the strength gains, while others propose the opposite. A previous study revealed that the concurrent training strategies (resistance + endurance / endurance + resistance) in young male individuals provided improvements in the VO_2max_ values of the participants, but these developments did not create a significant difference between the two training strategies [36]. In a meta-analysis, the results of 21 studies analyzing concurrent training and the interaction between its endurance and resistance components showed that concurrent training produces similar increases in maximal strength and VO_2max_ as with aerobic and resistance activities alone [38]. There are also different studies in the literature with results consistent with our research findings [39, 40]. A recent meta-analysis study examined the results of 19 randomized controlled trials. Finding showed lack of difference between strength + endurance and endurance + strength on VO_2max_ in the overall analysis [14]. In addition, it has been shown that exercise mode, period, frequency, and intensity and subject characteristics may influence the outcome of concurrent strength and endurance training [21, 22]. Thus, it’s possible that the volume of the training of twice a week of 13-week duration of the program may not have been long enough to elicit substantial changes in VO_2max_. Thus, participants’ dietary habits can also impact their fitness levels which required further investigation that was beyond the scope of this study. Additionally, utilizing direct measures of cardiopulmonary fitness, incorporating gas exchange analysis, could enhance the precision of our findings. In our current study, we relied on an indirect estimation of participants’ VO_2max_ based on parameters such as distance, body weight, and heart rate. We advocate for further research aimed at investigating the underlying physiological mechanisms, including both central and peripheral hemodynamic factors, that may elucidate the observed physiological responses.

The studies that were analyzed included participants from various age groups, populations, and with different characteristics, such as sedentary or non-sedentary individuals. Despite these differences, the analysis revealed that the exercise sequence did not affect the development of VO_2max_. However, it is important to note that the studies’ training methods were not taken into account during the analysis. This means that the interpretation of the results may not be accurate if the exercise methods were not considered. Therefore, it is crucial to examine the training methods of each study to better understand their results and implications. In other words, the way the participants exercised could have impacted their VO_2max_ levels, and this should not be disregarded when interpreting the study findings.

There was no significant difference in the groups’ FVC and FEV1 levels in terms of pulmonary function profile in the study. However, the PEF value increased significantly in ASG. No research has been found in the literature examining the effects of concurrent training methods on dynamic respiratory parameters in any population. Peak expiratory flow (PEF) is the maximum air flow rate obtained during a forced vital capacity maneuver and reflects the diameter of the central airways and the activity of expiratory muscles in healthy individuals. It is effort-dependent, like FEV1 [41]. Numerous studies examining the effects of both aerobic and resistance exercises on dynamic respiratory parameters have determined that both types of exercises have a positive effect on variables such as FVC, FEV1, and PEF [42–44]. Various training methods have been shown to have positive effects on respiratory function and respiratory muscle strength [45, 46]. Additionally, it has been reported that aerobic exercises have a positive effect on lung function [47]. Aerobic exercise primarily focuses on improving cardiovascular fitness, enhancing lung capacity, and optimizing oxygen utilization. During aerobic activities individuals engage in sustained, rhythmic movements that require continuous and controlled breathing which directly tend to promote greater lung expansion and strengthens the respiratory muscles over time, resulting in a positive effect on PEF. Conversely, strengthen exercise, including weightlifting or resistance band training, is primarily aimed at building muscle strength and endurance [48, 49]. While resistance exercise may not directly affect PEF to the same extent as aerobic exercise, it plays a vital role in overall pulmonary function. Strengthening the muscles associated with breathing can lead to improved respiratory function and coordination, indirectly contributing to enhanced PEF [50]. Finding in this study indicates that both strength and aerobic exercise types have shown improvements in PEF values. It has been found that a more effective exercise strategy for enhancing the PEF value is to train aerobic exercise after strength exercises.

The study revealed that participants in both the SAG and ASG groups experienced an increase in strength values, which were found to be at a similar level. This finding suggests that the order of exercise for strength development in middle-aged healthy individuals, whether before or after aerobic training, does not affect the level of strength development. It has been claimed that with increased endurance training, muscle strength, hypertrophy, and power will decline during concurrent training [14]. Endurance training improves the muscles’ oxygen utilization capacity by increasing cardiopulmonary function, myoglobin number, mitochondrial density and number, and aerobic enzyme activity. However, it is also accompanied by a decrease in the cross-sectional area of muscle fibers, which reduces the level of muscle strength or power [51]. In contrast to these research results, our study found that both training groups improved their strength levels. The differences in research results may be due to differences in training methods, differences in the age and gender of the participants, differences in the participants’ sports or exercise history, and changes in their fitness level. Specifically, Makhlouf et al. [45] found that strength training prior to endurance training could improve the dynamic strength of muscles more than the opposite training sequence. This may be due to endurance training leading to fatigue, which affects neuromuscular activation and reduces muscle firing frequency. Additionally, study by Wilhelm et al. [52] aligned with our findings and reported that regardless of the sequence of endurance and strength training, training was beneficial to enhance strength and power output in older adults. However, there was no significant difference between the groups in the comparison of the exercise sequence. Combined aerobic and low- or moderate-intensity resistance training increases muscle strength, regardless of the exercise order in older women [53]. The order of eight weeks of concurrent training does not significantly affect body fat percentage, physical fitness factors, and functional capacity of postmenopausal women [54].

The present study has some limitations. First, the fact that the participants’ nutrition and sleep patterns could not be controlled is also an important limitation. Furthermore, it is essential to acknowledge that the research groups exhibited an unequal ratio of male to female participants, which may limit the generalizability of the study findings.

## Conclusions

In conclusion, both training protocols resulted in significant improvements in participants’ body composition. Notably, there was a significant difference in the changes in PEF values between the two groups, with a decrease in the SAG group and an increase in the ASG group. These findings highlight the importance of considering the sequence of concurrent training when aiming to optimize body composition. The study demonstrates that a 2-day concurrent training program is a feasible option for adults, promoting their engagement and commitment to regular physical activity. These results hold practical implications for both individuals and fitness professionals, suggesting flexibility in designing exercise routines tailored to personal preferences, time constraints, or specific training objectives.

## Data Availability

(ADM) The data is not publicly available because further research is being done and more manuscripts are being prepared. Data for the current study will be available upon reasonable request from the principal investigator or corresponding author.

## Abbreviations

WHO: World Health Organization
PA: Physical activity
VO_2max_: Maximal oxygen uptake
IPAQ: International Physical Activity Questionnaire
SAG: Strength and Aerobic Group
ASG: Aerobic and Strength Group
RT: resistance training
CT: Concurrent training
